# Hybrid Process Chain for the Integration of Direct Ink Writing and Polymer Injection Molding

**DOI:** 10.3390/mi11050509

**Published:** 2020-05-18

**Authors:** Dario Loaldi, Leonardo Piccolo, Eric Brown, Guido Tosello, Corey Shemelya, Davide Masato

**Affiliations:** 1 Department of Mechanical Engineering, Technical University of Denmark, 2800 Kgs. Lyngby, Denmark; darloa@mek.dtu.dk (D.L.); guto@mek.dtu.dk (G.T.); 2 Department of Plastics Engineering, University of Massachusetts Lowell, Lowell, MA 01854, USA; leonardo_piccolo@uml.edu; 3 Department of Electrical and Computer Engineering, University of Massachusetts Lowell, Lowell, MA 01854, USA; Eric_Brown2@student.uml.edu (E.B.); Corey_Shemelya@uml.edu (C.S.)

**Keywords:** direct-writing, additive manufacturing, injection molding, micro manufacturing, functionalization

## Abstract

The integration of additive manufacturing direct-writing technologies with injection molding provides a novel method to combine functional features into plastic products, and could enable mass-manufacturing of custom-molded plastic parts. In this work, direct-write technology is used to deposit conductive ink traces on the surface of an injection mold. After curing on the mold surface, the printed trace is transferred into the plastic part by exploiting the high temperature and pressure of a thermoplastic polymer melt flow. The transfer of the traces is controlled by interlocking with the polymer system, which creates strong plastic/ink interfacial bonding. The hybrid process chain uses designed mold/ink surface interactions to manufacture stable ink/polymer interfaces. Here, the process chain is proposed and validated through systematic interfacial analysis including feature fidelity, mechanical properties, adhesion, mold topography, surface energy, and hot polymer contact angle.

## 1. Introduction

Recent research efforts have opened new opportunities for the integration of Additive Manufacturing (AM) technologies with other mass-market fabrication techniques. The increasing industrial adoption of AM technologies has been shifting the focus from prototyping to direct integration with established processing technologies and applications. In particular, direct-write additive manufacturing has shown promise in a variety of applications ranging from radio frequency (RF) to sensing and even microfluidic integration [1,2,3,4,5,6,7,8,9,10]. Among AM technologies, direct-write micro-dispensing using silver conductive inks is one of the most common methods used to create printed electronics [11,12]. However, direct-write applications are no-longer limited to printed electronics, and inks are no-longer limited to silver-based nano particles. For example, Ruthenium and Barium Strontium Titanate inks have been developed for dielectric properties, Li4Ti5O12 (LTO) and LiFePO4 (LFP) have been used for printed batteries, BO_3_Y (yttrium borate) was used as a printable luminescent material, and even bio-materials including silk fibroin has been printed with direct-write methods [13,14,15,16,17,18,19,20,21,22,23,24,25,26]. The material and process flexibility of direct-write AM offers significant opportunities for integration with mass-manufacturing technologies, such as plastic injection molding.

The current integration of functional features into plastic parts relies on the use of multi-step technologies, such as insert molding [27] or injection over-molding [28]. These technologies are characterized by high productivity, but limited flexibility for the integration of electronic features and functionalities. High production volumes are guaranteed through the separation of manufacturing in two consecutive steps: injection molding of the device and fabrication of the circuit traces.

Recent efforts to improve design flexibility have focused on post-processed methods. In particular, new process chains have been proposed to integrate printed circuits on injection molded parts using direct-write onto the plastic surface [29]. However, typical direct-write printing would require printing on difficult-to-reach areas, such as slots or internal surfaces to create complex geometries [30]. Moreover, the surface roughness, structures adhesion, and mechanical resistance of AM circuits can be critical factors in some applications, such as RF and sensing.

Here we propose the use of direct-writing AM technology to additively deposit conductive features on an injection mold surface. The printed traces undergo a transfer process into the polymeric part during the molding process. The volumetric integration of printed features into plastic parts is enabled by controlling the relative plastic/ink and mold/ink interfacial strengths. This process can also be further enhanced through the use of engineered injection-mold surface properties and coatings which have previously been demonstrated as an effective solution to control polymer flows both during the injection [31,32] and ejection phase [33].

To understand the printing of functional ink features onto injection mold materials, it is crucial to understand the interface interactions involved in the process. The creation of a quality ink/mold interface is the key parameter to achieve consistent printing. However, the ink/polymer interface should be stronger, to enable the final “transfer” to the plastic part. The ink/polymer interface is controlled by a variety of phenomena, including surface roughness [34], surface energy [35,36], and processing conditions [37]. In particular, high surface roughness has been shown to promote stronger interfacial interactions [38]. For these high roughness interfaces, the polymer melt has the capability of entering topographical voids, increasing the surface contact area between the melt and mold [39]. The investigation of these interfaces should also consider the differences in surface energy (i.e., polar and a-polar components) between two materials. The design of the material systems involved at the interfaces, allows achievement of stronger or weaker surface adhesion, depending on the similarities between polar and a-polar components of materials surface energy [40].

In this work, a hybrid molding process chain that enables the combination of ink dispensing technologies with conventional plastic injection molding is developed. Here we describe how nano-particle ink is printed, cured, and sintered before exposure to the polymer melt. After molding, the ink system of traces is embedded in the polymer parts and released from the mold surface due to a customized ink/polymer interface with differential polymer shrinkage. This work reports the characterization of the interactions at the mold/ink/polymer interfaces considering topography parameters, surface energy, and contact angle of the hot polymer melt. Additional mechanical testing is performed to evaluate the strength of molded devices and the adhesion of ink traces to the plastic parts produced with this method. It is expected that the advantages of integrating the embedded traces during molding will enable a stronger ink/polymer interface and improve surface homogeneity and finish compared to post-process printing methods.

## 2. Process Chain Development

**Figure 1:**
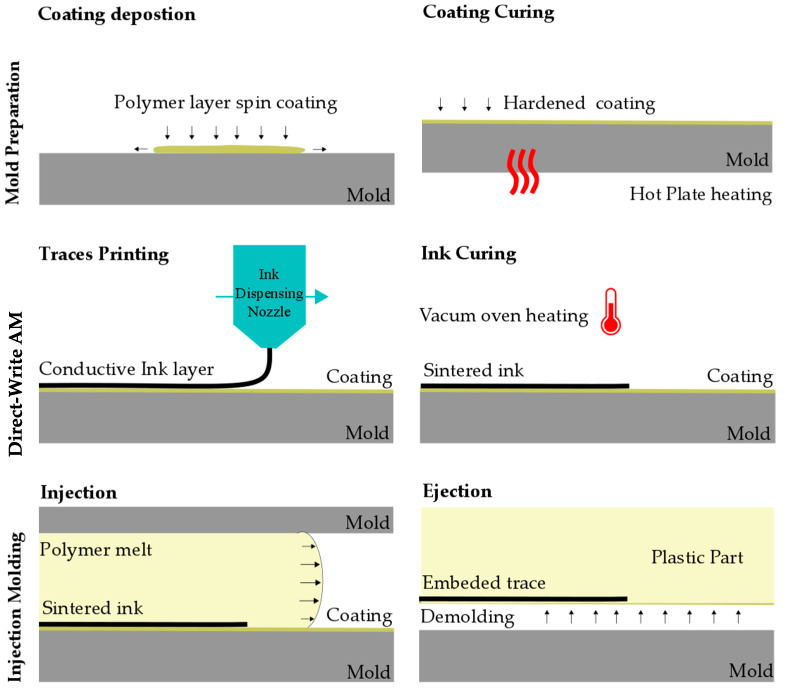
Steps of the process chain developed to manufacture molded interconnect devices.

Figure 1 describes the novel process chain and its main manufacturing steps. The process chain follows three primary processing steps: (i) mold preparation, (ii) direct-write AM, (iii) injection molding.

(i) Mold preparation: the mold was first pre-treated using a solvent/polymer solution, which was deposited on the surface where the ink will be printed. The releasing agent ensures high-quality printing and improves release to the polymer system during the injection molding process.

(ii) Direct-write AM: the printing process was carried out using an ink micro-dispensing system (Pro4, Nordson EDF, East Providence, RI, USA). The dispensing nozzle can be adjusted for multiple trace dimensions with the resulting feature sizes dependent on the selected ink, the deposition rate, and curing methods. Once printed, the ink was cured/sintered in a vacuum oven. It should be noted that other curing methods could be used including laser curing and photonic curing.

(iii) Injection molding: the mold, with the printed/sintered traces, was assembled into an injection molding machine for processing. The injected polymer melt flowed over the structures and the rapid cooling formed a strong bond between the melt and ink. The final polymer part with integrated traces was then ejected from the mold, resulting in a hybrid, composite part.

## 3. Materials and Methods

### 3.1. Mold Surface Preparation

Before printing conductive ink onto the injection mold surface, the mold was pretreated with an ABS (Trilac^®^ ABS-MP1000 Polymer Technology and Services, LLC (PTS), Heath, OH, USA) solvent solution. A typical coating process would include solution-based spin coating, alternatively, spray coating techniques to compensate for mold size and/or weight constraints.

The resulting mold surface maintains a thin, uniform, polymer coating directly on the stainless-steel. After coating, the mold is heated on a hot plate at 120 °C for 5 min to remove excess solvent from the coating. The surface treatment was designed and used to improve relative surface energies, enabling optimal mold/ink interface for molding “transfer.”

### 3.2. Direct-Write Printing

A silver nanoparticle ink (CB 028, DuPont^®^, Wilmington, DE, USA) was selected for testing as it represents the standard ink-based material system in the field of printed electronics. As such, this investigation also represents a general proof-of-concept for eventual process integration for electrical applications.

**Figure 2:**
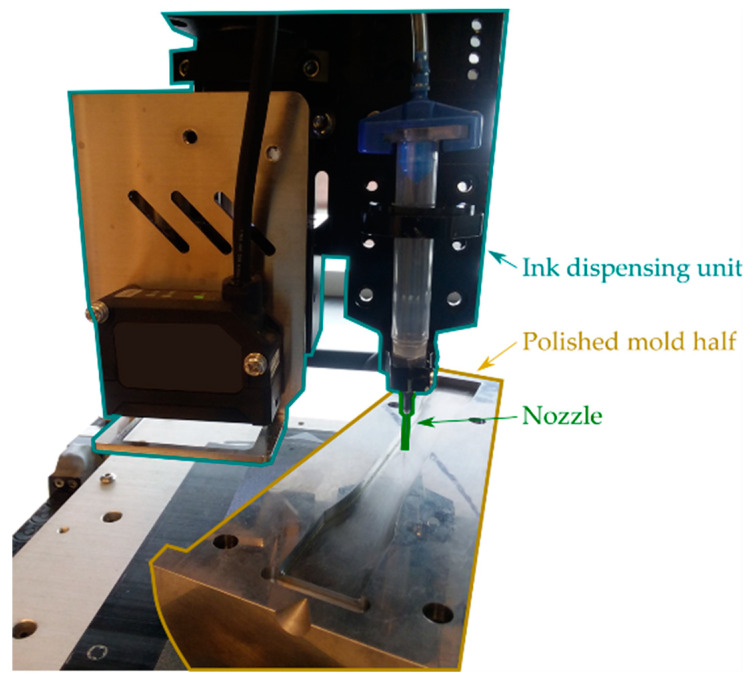
Ink dispensing system before printing over the injection mold surface.

Printing was carried out on the pre-treated mold surface using an automated micro-pen dispensing system (Nordson Pro4 EFD). Figure 2 shows a mold inside of the Nordson Pro4 EFD before printing. After printing, the mold with the printed traces was placed in a vacuum oven (Isotemp 282A, Thermo Fischer Scientific, Waltham, MA, USA) for sintering at 220 °C for 30 min.

To evaluate the effects of polymer pressure on ink/polymer interlocking and adhesion, printed traces were located at specific locations of the mold surface, based on the relative distance to the injection location. Each trace was printed in the form of a line with a design length of 20 mm, and a design height of 50 μm.

### 3.3. Injection Molding Setup

Injection molding tests were run on a micro injection molding machine (Xplore^®^ IM 12, Xplore Instruments BV, Sittard, The Netherlands). The machine features an injection cylinder of 10 mm in diameter with a maximum shot volume of 12 cm^3^. The injection molding process conditions were set as follows: mold temperature at 80 °C, melt temperature at 260 °C, holding time of 8 s at 0.7 MPa. A single cavity stainless-steel mold was used to manufacture ASTM D638-14 type I tensile bars. During injection molding experiments, the mold with the printed traces was mounted on the machine and removed after every cycle for cleaning and additional printing.

The resin used for the experimentation was acrylonitrile butadiene styrene ABS (Trilac^®^ ABS-MP1000 Polymer Technology and Services, LLC (PTS), Heath, OH, USA). This polymer was selected for its good processability, low viscosity, and rigidity. Additionally, this ABS resin is compatible with the coating layer that was used for mold pre-treatment before ink deposition.

### 3.4. Imaging and Topography Characterization

**Figure 3:**
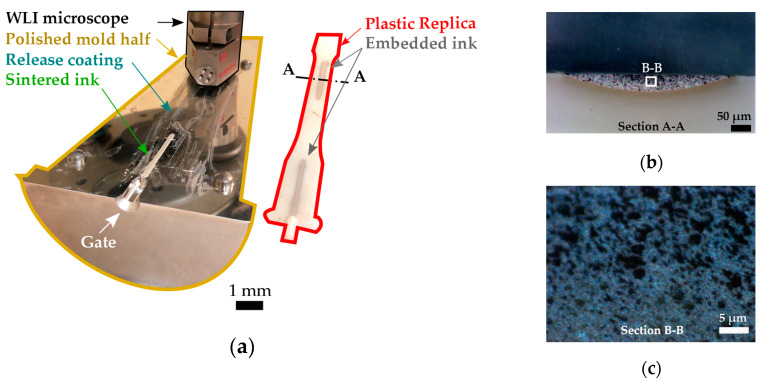
(**a**) Characterization of the mold before injection and the plastic replica after transfer. (**b**) Cross-section of the plastic part with the embedded traces, and (**c**) high magnification of the ink embedded in the printed traces.

The dimensions of the traces printed on the mold surface and their qualitative inspection were performed using a stereomicroscope (SteREO Discovery V20, Zeiss, Oberkochen, Germany) to scan the whole printing area. Topographies were acquired using an optical profiler (Wyko NT2000, Bruker Nano, Tucson, AZ, USA), using a 20x magnification lens equipped with a Mireau interferometer. Figure 3a shows the characterization setup and the areas that were scanned.

From the acquired topographies, surface roughness *Sa* and mean summit curvature *Ssc* were evaluated according to ISO 25178:2012-2. These areal parameters were compared at multiple locations. The surface amplitude roughness *Sa* was used as a quantitative indicator of amplitude while the average surface summit curvature *Ssc* characterizes the contact area between multiple surfaces.

The molded parts were sectioned to evaluate interface adhesion and morphology. Samples were embedded in a thermoset resin for polishing. For a smooth interface, the polishing process (Ecomet 250, Buehler, Lake Bluff, IL, USA) consisted of alternating pans of increasing mesh up to 3,000 with a total processing time of 20 min. Figure 3b shows a cross-section of a molded part with the integrated printed ink trace. The interface surfaces were qualitatively analyzed using Scanning Electron Microscopy (SEM - FEI, Quanta 400, Thermo Fisher Scientific, Waltham, MA, USA), allowing identification of voids, interface interlocking, and overall ink/polymer adhesion.

### 3.5. Surface Energy and Polymer Melt Contact Angle

The adhesion at the ink/mold and ink/polymer interfaces was evaluated by quantifying the surface energy for the different materials involved in the process chain. Surface energy was measured using a drop shape analyzer (DSA 100, Krüss GmbH, Hamburg, Germany). Two different liquids (water and diiodomethane) were used for the estimation of surface energy based on the extended Fowkes’ two liquids in contact model [41]. The measurements were performed on the following substrates: mold-polished steel, mold steel pre-treated for printing, sintered ink, and ABS molded parts in a trace-free area.

The effect of injection molding on the creation of a strong polymer/ink interface was evaluated by measuring the contact angle of the hot polymer melt over the mold surface. These tests were performed using the same drop shape analyzer on which a high temperature syringe dosing unit (TC21, Krüss GmbH, Hamburg, Germany) and measuring cell (TC3213, Krüss GmbH, Hamburg, Germany) were mounted. Contact angle measurements were performed using printed/sintered ink and hot ABS at injection molding melt temperature (i.e., 260 °C). The measurements were performed on polished steel.

### 3.6. Mechanical Properties 

Mechanical properties of the injection molded parts with and without embedded traces were evaluated using standard ASTM dog-bone samples. The mechanical properties of the base ABS were benchmarked on ten samples manufactured following the procedure described in Section 3.3. Additionally, the mechanical properties were characterized for five samples manufactured with the proposed process chain.

Tensile testing (5966, Instron, Norwood, MA, USA) was carried out at room temperature according to ASTM D638. A load of 50 kN was used to achieve plastic fracture and the respective stress-strain curve. Young’s modulus was calculated as the ratio of the stress at a percent strain of 0.2 %, while the Ultimate Tensile Strength (UTS) was evaluated as the maximum stress value before breakage.

### 3.7. Peeling Test 

Further analysis of the molded parts with the integrated traces was carried out through a peeling test. The adhesion strength of the ink/plastic interface was evaluated according to ISO 2409:2013 cross-cut test. The test was performed using a hand-held, multi-blade cutting tool with a 1 mm cutting spacing between each blade. A pressure-sensitive adhesive tape (12.7 mm width, 600 HC-33 from 3M^®^) was used to remove the loose ink. The embedded ink trace were determined to have height of 200 µm, width of 2 mm, and length of 6 mm. The test consisted of sectioning the printed traces, attaching the tape to the surface of the hybrid part, and peeling. The result of the test is evaluated by inspecting the portions of the printed ink that are removed with the tape.

## 4. Results and Discussion

### 4.1. Influence of Surface Roughness

**Figure 4:**
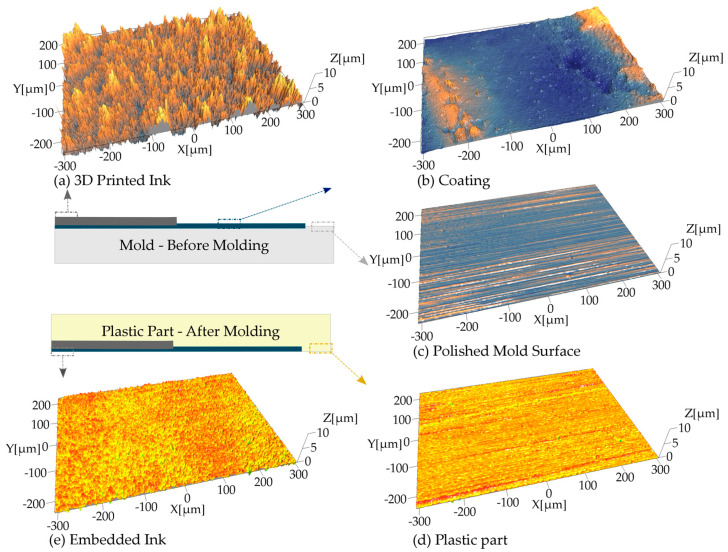
3D view of the analyzed surface topographies: (**a**) 3D printed ink on the mold before injection molding, (**b**) coating on the mold before injection molding, (**c**) polished mold surface, (**d**) plastic part surface after demolding, (**e**) embedded ink on the plastic part.

Figure 4 compares the measured surface features over the different surfaces described in the process chain. The results of the surface roughness evaluation showed significant differences between the mold, the release coating, and the sintered ink and are described in Table 1.

The printed ink surface had the highest roughness and summit curvature (cf. Figure 4a). Compared to the polished side of the mold, the ink shows a *Sa* higher by a factor 35x and an *Ssc* higher by a factor 115x. This is typical of direct-writing and AM technologies and is due to the particle-based nature of the inks. These traces are usually characterized by high surface roughness and their topography often exhibits inhomogeneity and macroscopic defects [42]. As shown in Figure 4e, embedding the traces during the molding process resulted in an average *Sa* value of 315 nm, which is 4x smaller than the printed ink trace alone (cf. Figure 4a). This roughness reduction confirms the effectiveness of the process chain in reducing the surface roughness of the printed electronics in the plastic part. Therefore, by printing the traces onto the mold and then transfer them via injection molding, it is possible to embed printed interconnects within a plastic part.

**Figure 5:**
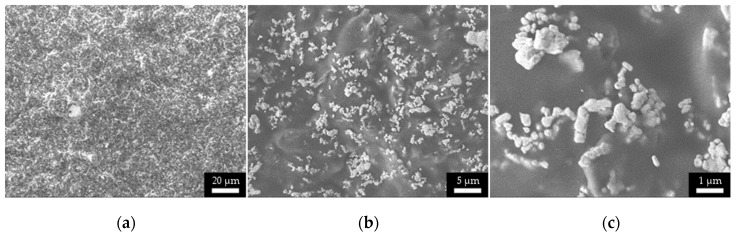
SEM micrographs at different magnification (500× (**a**) –3000× (**b**) –12000× (**c**)) of the sintered ink surface before injection molding. Micrographs were taken with an E beam power of 5 kV.

**Figure 6:**
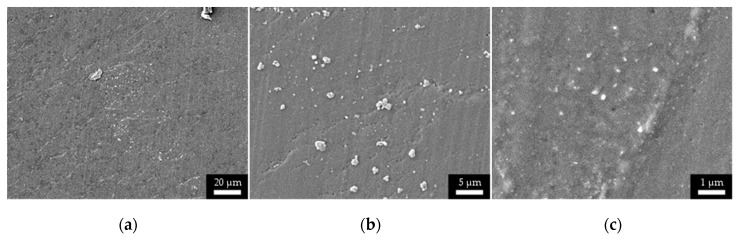
SEM micrographs at different magnification (500× (**a**) –3000× (**b**) –12000× (**c**)) of the sintered ink surface embedded in the injection molded part. Micrographs were taken with an E beam power of 5 kV.

The SEM micrographs (Figure 5) of increasing magnification (500×–3000×–12000×) display the anisotropic nature and high roughness of the sintered ink surface before molding. As a comparison, Figure 6 shows the smoother topography resulting from the hybrid process chain presented in this work. Indeed, the traces printed on the injection mold surface are “flipped” when transferred to the plastic system in the injection molding process. The “transfer” process has the advantage of creating a smoother outer surface because of volumetric integration. This volumetric integration also is expected to result in higher wear resistance and higher resistance to mechanical shears.

Moreover, the high initial roughness of the printed traces, facing the polymer melt, favors interlocking upon injection molding. The molten polymer fills these topographical voids creating strong interactions at the polymer/ink interface. This exploits the high surface roughness typical of AM parts to generate higher interface adhesion and guarantees functionality.

### 4.2. Surface Energy

**Figure 7:**
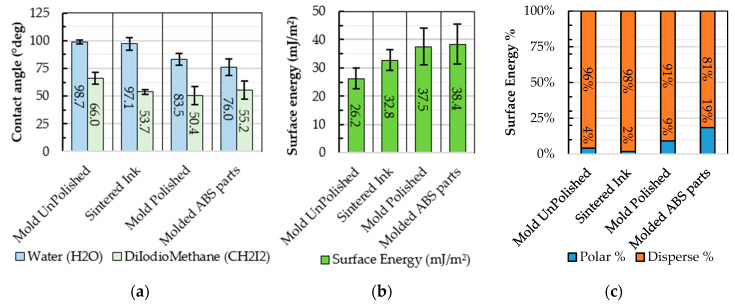
(**a**) Contact angle measurements for polar (H_2_O) and a-polar (CH_2_I_2_) liquids: (**b**) surface energy for different surfaces; (**c**) percentage of dispersion and polar content for the analyzed surfaces.

The surface energy of the different interfaces was used as an indirect measure of the adhesion strength between different materials interfaces [35]. By using contact angles measurements with a-polar liquid (water) and a-polar liquid (diiodiomethane) it was possible to calculate the total surface energy using the Fowkes’ model [41]. From the contact angle measurements with the two liquids (Figure 7a), surface energy for different surface were obtained (Figure 7b). Then, the percentage of polar or disperse energy (Figure 7c) were analyzed to evaluate surface interactions.

Considering the experimental results, it was observed that surface energy increases significantly when reducing the surface roughness. In particular, the polished mold surface revealed a surface energy composed of 91% disperse and 9% polar contributions, which is double the polar affinity of an unpolished specimen. Similarly, the ink surface after curing is characterized by higher dispersion content, equal to 98% but now only a 2% polar contribution. Moreover, the overall surface energy in the polished mold surface is higher than the sintered ink, increasing from 32.8 ± 3.8 mJ/m^2^ to 37.5 ± 6.5 mJ/m^2^.

**Figure 8:**
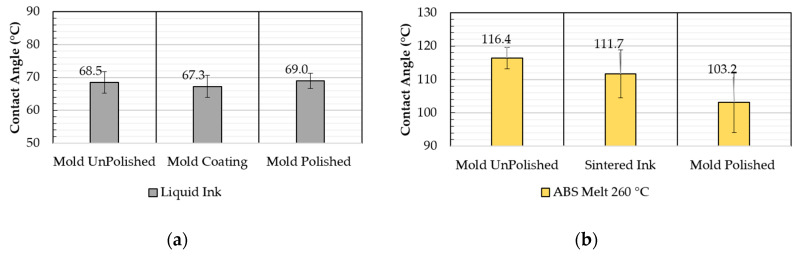
Contact angle measurements using (**a**) liquid ink and (**b**) ABS melt at 260 °C on different surfaces.

Contact angle measurements using the liquid ink (Figure 8a), suggest that the mold pre-treatment, used for release after injection molding (cf. Section 3.1), does not influence the direct-writing process, and thus the quality of ink deposition. However, the analysis of surface energy shows that higher surface affinity is formed between the mold and the plastic melt as opposed to the plastic and the sintered ink. From a process chain perspective, this measured surface affinity is very important to understand why the mold pre-treatment (cf. Section 3.2) favors the release and transfer mechanism [43]. In the absence of the pre-treatment layer, the ink would have a higher affinity with the mold steel, and transfer upon injection molding would not occur.

Contact angle measurements of the hot polymer melt were performed using a heated chamber. ABS samples were heated at 260 °C (i.e., molding temperature) and deposited on different surfaces to study the wetting behavior. The results indicate that a higher contact angle is formed over the sintered ink (i.e., 111.7°) and the unpolished mold surface (i.e., 116.4°) when compared to the polished mold surface (i.e., 103.2°). This contact angle adjustment demonstrates the effect of surface roughness on polymer/mold surface affinity, which is high for the sintered ink. As reported by Sorgato et al. the wettability of the polymer to a specific mold surface is crucial to determine the capability of the polymer to replicate the topography [36].

### 4.3. Mechanical Integrity and Interface Strength

**Figure 9:**
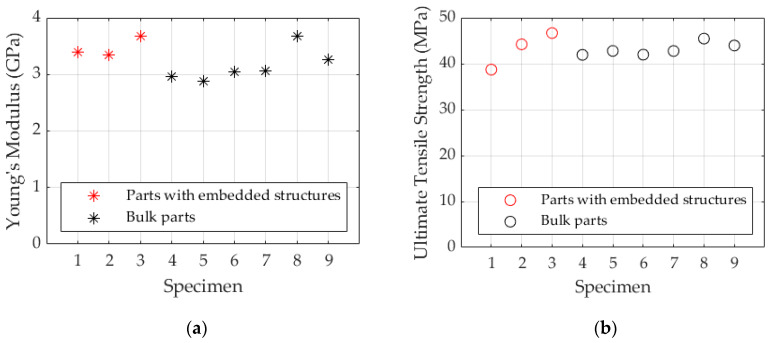
(**a**) Elastic modulus and (**b**) Ultimate Tensile Strength for samples with embedded printed electronics and bulk parts.

The Young’s modulus of elasticity, measured at 0.02% of elongation, and the Ultimate Tensile Strength (UTS) were used to analyze the effects of trace inclusion on the mechanical strength of the composite parts. The results are shown in Figure 9a,b, respectively. The average Young’s modulus of the traditional parts is 3.1 ± 0.3 GPa, and the parts with embedded structures show a Young’s modulus of 3.5 ± 0.2 GPa. This slight difference suggests that the embedded printed traces may act as a reinforcement, increasing the stiffness of the plastic part.

Analysis of the UTS values suggests that the printed traces embedded in the parts have no significant effect on the overall mechanical strength. The average for the ABS parts is 43.1 MPa, while the parts with the embedded traces have an average UTS of 43.2 MPa. This consistent UTS indicates that integration of the traces does not introduce any points of high-stress concentration, and the hybrid process chain has no negative effect on the tensile strength of the hybrid part.

**Figure 10:**
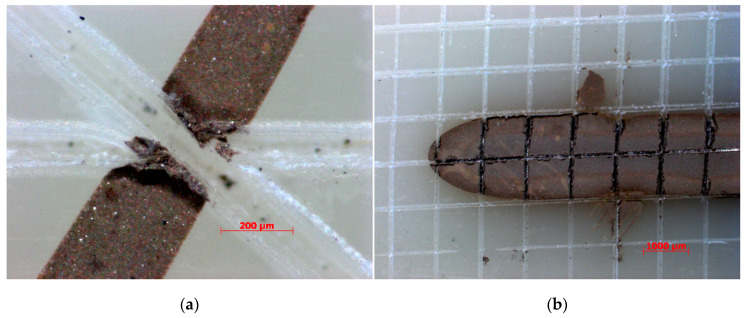
Optical images of the embedded traces after the peeling test for a trace with a nominal width of (**a**) 250 µm and (**b**) 2000 µm.

The adhesion at the ink/polymer interface for the parts was evaluated by carrying out peeling tests (Figure 10). The test was conducted on multiple traces located at multiple distances from the injection location, as described in Section 3.2. For all locations, strong adhesion was observed and no noticeable loss in adhesion could be measured. The results indicate that the proposed process chain can create consistent adhesion along the whole cavity where the polymer pressure is different.

## 5. Conclusions

This work presented an innovative process to integrate printed silver traces into injection molded plastic parts. This manufacturing process can be used to produce plastic parts that embed printed silver traces with custom surface roughness, unaltered mechanical properties, and strong polymer/electronic adhesion. The experimental results have demonstrated the effectiveness of the process in creating mechanical interlocking between the polymer system and the printed electronics.

The main findings of this work include:

- Topographical analysis of the different surfaces to verify the proposed process when compared to printing directly onto molded parts as a post-process. Specifically, the surface roughness of parts with integrated traces was reported to be low compared to that of post-processed, printed structures. The improved surface finish is explained by the intrinsic rotation of the structures during the “transfer” process.
- The high surface roughness of the printed structure before molding was exploited to create favorable interlocking at the ink/polymer interface, increasing adhesion. The trace transfer process from the mold/ink interface to the ink/polymer interface is enabled by exploiting the similar surface energy between the materials. Specifically, the results demonstrated that the surface energy affinity at the ink/polymer interface must be stronger than that of the ink/mold interface to promote transfer, indicating the need for a pre-treatment coating.
- It was expected that the composite molded parts would retain the mechanical stability, of bulk ABS parts. Our statistical analysis verified this effect, where volumetric integration of the printed traces into the plastic does not affect their structural properties. Moreover, peeling tests and micrographs of cross-sections demonstrate sufficient adhesion strength between the polymer and the ink.
- This work presents a first-in-class study on the feasibility, and methods required to integrate, additive direct-write tools with injection molding. The result is a hybrid technique enabling the fabrication of custom, design-on-demand plastic parts with integrated conductive structures. Future work will focus on the integration of additional additive techniques and the integration of more complex structures and molding techniques.

## Figures and Tables

**Table 1:** Summary table indicating *Sa* and *Ssc* parameter results for the analyzed topographies.

Surface	3D Printed Ink	Coating	Polished Mold Surface	Plastic Part	Embedded Ink
*Sa*/nm	1396	603	39	52	315
*Ssc*/µm	1.15	0.10	0.01	0.02	0.19
